# Towards an Inclusive and Evidence-Based Definition of the Maternal Mortality Ratio: An Analysis of the Distribution of Time after Delivery of Maternal Deaths in Mexico, 2010-2013

**DOI:** 10.1371/journal.pone.0157495

**Published:** 2016-06-16

**Authors:** Hector Lamadrid-Figueroa, Alejandra Montoya, Jimena Fritz, Marisela Olvera, Luis M. Torres, Rafael Lozano

**Affiliations:** 1 National Institute of Public Health, Cuernavaca, Morelos, México; 2 Independent collaborator, Mexico City, México; 3 Institute for Health Metrics and Evaluation, Seattle, WA, United States of America; BRAC, BANGLADESH

## Abstract

Progress towards the Millennium Development Goal No. 5 was measured by an indicator that excluded women who died due to pregnancy and childbirth after 42 days from the date of delivery. These women suffered from what are defined as late deaths and sequelae-related deaths (O96 and O97 respectively, according to the International Classification of Diseases, 10^th^ revision). Such deaths end up not being part of the numerator in the calculation of the Maternal Mortality Ratio (MMR), the indicator that governments and international agencies use for reporting. The issue is not trivial since these deaths account for a sizeable fraction of all maternal deaths in the world and show an upward trend over time in many countries. The aim of this study was to analyze empirical data on maternal deaths that occurred between 2010 and 2013 in Mexico, linking databases of the *Deliberate Search and Reclassification of Maternal Deaths (BIRMM)* and the *Birth Information Subsystem* (SINAC) of the Ministry of Health. Data were analyzed by negative binomial regression, survival analysis and multiple cause analysis. While the reported MMR decreased by 5% per year between 2010 and 2013, the MMR due to late and sequelae-related deaths doubled from 3.5 to 7 per 100,000 live-births in 2013 (p <0.01). A survival analysis of all maternal deaths revealed nothing particular around the 42 day threshold, other than the exclusion of 18% of women who died due to childbirth in 2013. The multiple cause analysis showed a strong association between the excluded deaths and obstetric causes. It is suggested to review the construction of the MMR to make it a more inclusive and dignified measurement of maternal mortality by including all deaths due to pregnancy and childbirth into the Maternal Death definition.

## Introduction

The World Health Organization defines maternal deaths as *"deaths of women while pregnant*, *during childbirth or within 42 days of termination of pregnancy*, *irrespective of its duration and site of occurrence*, *and as a result of any cause related to or aggravated by the pregnancy or its management but not from external causes (homicides*, *suicides and accidents unrelated to medical care)"*.[1] The definition of what constitutes a maternal death is crucial for its measurement, since the definition of the Maternal Mortality Ratio (MMR) naturally depends on it. According to the manual used to measure the progress of Millenium Development Goal (MDG) #5, the MMR only takes direct obstetric causes (International Classification of Diseases 10th revision [ICD-10]: O00-095) and indirect obstetric causes (ICD 10thO98-099) into account, leaving out late maternal deaths (O96) and the sequelae of direct obstetric causes (O97).[2] Late maternal deaths are defined by ICD-10 as *"the death of a woman from direct or indirect obstetric causes after 42 days but within one year of the termination of pregnancy"*; maternal deaths from direct obstetric causes’ sequelae are in turn defined as "*deaths from any obstetric cause occurring one year or more after delivery*." [1,3] Beyond the arbitrariness of placing a threshold at 42 days that is not justified by scientific evidence, the exclusion that results from it is discriminatory for scores of women who died because of their motherhood, as tragically as those that were indeed counted.[4]

The specialized literature on maternal mortality speaks little about the subject of late deaths and sequelae; an exception is the study published by Kassebaum et al, which presents trends in late and sequelae-related deaths.[5] This study estimates a drop of its MMR of 34–31 per 100,000 live births between 1990 and 2013, which translates into a 9% lower risk over a period of 23 years. However, there is a striking heterogeneity in the trends; countries as China, India and Cuba have decreasing late maternal death ratios while in contrast, countries such as USA, Canada, Brazil and South Africa show an increase.[5]

Mexico, a country with good maternal mortality data, has shown a significant progress towards the MDG #5. According to the Mexican government and two years before the closure of the period involved, the country managed to reduce the Maternal Mortality Ratio by 57% from the year 2000, which is an improvement of 76% from 1990–2013.[6] However, Mexico is one of the countries where late maternal deaths have actually increased.[5] The fact that late maternal deaths are increasing in several large countries, a trend that diverges from the decrease in the official MMR, makes us wonder about whether we are truly avoiding maternal deaths, or only postponing them.

Undoubtedly, the information yielded by the analysis of late maternal deaths and deaths due to sequelae is of great interest to maternal health specialists and particularly for those responsible for operating the maternal health programs at different levels of the health system. The exclusion of these deaths for not being “statistically useful” for calculating the MMR as indicated by the United Nations manual, far from helping, hinders our understanding of the phenomenon of maternal deaths and hence the approach to effective and fair preventive measures. By contrast, the analysis of the totality of the maternal deaths, will allow us to understand the problem as a whole, extending our range and allowing us to act with the same energy regardless of them happening within the first six weeks or within the first year.

The objective of this work was to analyze the distribution of survival time elapsed from the end of pregnancy to death of women who died due to pregnancy and childbirth related causes in Mexico, as well as ascertaining causes related to late and sequelae-related deaths, during the 2010–2013 period using data from the official Mexican Health Information Systems.

Beyond proposing a parallel reporting of late and sequelae-related maternal deaths, we will use the results we present henceforth to portray the need to amend the existing definition, in a way that a large majority of maternal deaths are indeed included in the numerator of the MMR.

## Methods

### Data sources

Information on deaths was obtained from the databases of the *Deliberate Search and Reclassification of Maternal Deaths* (BIRMM, acronym in Spanish) generated by the General Directorate of Health Information of the Mexican Ministry of Health (DGIS).[7,8] Information on births was obtained from databases compiled by the Mexican *Birth Information Subsystem* (SINAC), a comprehensive database of all birth certificates in the country.[9] In broad terms, the BIRMM process researches documentation and confirms maternal death cases based on analyses conducted by technical committees which collect data from available sources; including death certificates, medical records, verbal autopsy records and confidential enquiry or autopsy reports. One of the outputs of this process is an *ad hoc* very brief clinical summary or case history in text form, typically between 50 and 100 words, which in practice may or may not include information about the end of pregnancy (explicit date, or mention of the number of days, months or years elapsed between the end of pregnancy and death.) The complete BIRMM process, along with its strengths and limitations has been described in detail elsewhere.[8,10]

In order to link these two datasets, we first improved the quality of the datasets by redistributing missing and unspecified data using Multiple Imputation by Chained Equations. [11] Complete datasets of deaths (from BIRMM) and births (from SINAC) originally at individual level were then aggregated into years (2010 to 2013). Then, we merged the aggregated databases matching them by year; the result is a contingency table that contains the count of deaths (by all maternal causes and divided by major group causes, including late and sequelae-related), and births, allowing us to calculate the MMR for each year and to analyze it using a count data model (see Statistical Analysis).

### Calculation of days elapsed between the end of pregnancy and death

Because the days elapsed between the end of pregnancy and death are not reported as an explicit variable in death certificates, it was necessary to extract information from the BIRMM’s clinical summaries. We considered the following information as relevant for calculation: the date of the end of pregnancy, defined as the date of vaginal delivery, cesarean section, stillbirth or abortion, and in its absence, explicit mention of days, months or years elapsed between the end of pregnancy and death.

Retrieval of information from the BIRMM clinical summary was done in two stages. The first stage focused on a process of manually extracting information conducted by three observers, who were trained by two expert researchers on how to single out and extract information from the clinical summaries through identifying key aspects. The training ended with a pilot phase, where the observers independently reviewed clinical summaries corresponding to the year 2013, and retrieved data on end of pregnancy dates, reaching an inter-observer Pearson’s’ correlation between 0.93 and 0.98 (the analyst was blinded to the identity of the observers). Additionally, they were asked to identify and report regularities: key words and phrases that were frequently repeated and that facilitate the location of relevant information within the summaries (examples of regularities found in the text are expressions such as: “parto el dd/mm/aaaa” [delivery on dd/mm/yyyy]; “parto ## días antes” [delivery ## days before]; “fallece ## meses después” [dies ## months after]). In the second stage, an automated text-encoding algorithm was defined, fed by information on observed regularities reported by the observers.[12] When the algorithm was unable to detect regular expressions in the clinical summary, we used the information manually extracted by the observers, if any.

There was a vast heterogeneity in the way information on the timing of the end of pregnancy was reported in the clinical summaries. In some cases, the exact date of which pregnancy ended was reported, in other cases the exact number of days elapsed was found in the summary; however on many occasions only vaguer information such as mention of months or years elapsed (phrases such as *“…death occurred two months after delivery…”*) was available. Thus, we devised the following procedure to estimate the elapsed dates according to the available information on the summary: When either the algorithm or the observers managed to retrieve the exact date of the end of pregnancy from the BIRMM clinical summaries, the number of days elapsed was obtained simply by counting the days to the date of death; this happened in 17.6% of the cases. When the actual number of days elapsed was retrieved, these were directly used; this occurred in 47.5% of the deaths. If only information about the elapsed months were available (1.5%), the exact days were assigned by multiplying n-1 months times 30.5, and then adding a uniformly distributed random number between 1 and 31. When only information about the elapsed years was available (1.4%), days were assigned as the multiplication of n-1 years times 365 plus a uniformly distributed random number between 1 and 365. If no information on date, days, months or years was identified, but when the summary indicated death occurred during the immediate postpartum period, a survival time of 1 day was assigned; if the summary mentioned death occurred during the postpartum period, a random number of days was assigned; the number took values between 1 and 10 when the summary mentioned death occurred during the subacute postpartum period, 11 to 42 when the summary mentioned death during the delayed post-partum period, and 1 to 42 days if the summary only mentioned death occurred “*during the postpartum period*”. The assigned values for this 2.2% of the cases were generated taking the integer of the exponential of a uniformly distributed random number between the natural logarithms of the lower and upper limits of each period. For example, for the subacute postpartum period we assigned a number between 1 to 10 by choosing randomly a number between ln(1) = 0 and ln(10) = 2.30; if the number chosen were 1.2 then the assigned value would be 3, the integer of its exponential: exp(1.2) = 3.38. A total of 17.4% of the deaths were not included in the analysis as absolutely no information on the time elapsed between the end of pregnancy and death was recovered. Finally 12.4% of the registered deaths occurred before the end of pregnancy.

Although the clinical summaries from years 2010–2012 are also available in the BIRMM dataset, for the analysis of days elapsed after the end of pregnancy we decided to only analyze data from 2013 as it best represents the current state of affair regarding late maternal deaths in Mexico.

### Statistical analysis

To obtain estimates for the official MMR and late and sequelae-related MMR, as a simple function of time, we fitted negative binomial regression models where the dependent variable was the number of maternal deaths (MM) and the denominator was the number of births (N) occurred in each year of interest (2010 to 2013) *t* of the time variable, denoted as T. Time (T) was the sole independent variable of the model.

$$\begin{array}{l} \text{log}\left(\frac{MM_{t}}{N_{t}}\right)=\beta_{0}+\beta_{1}T_{t}+\varepsilon_{t} \end{array}$$ (1)

Although our original choice of model was Poisson, the most-often used approach for count variables, examination of the data showed evidence of overdispersion (excess variance). As an alternative we decided to fit negative binomial regression, which takes overdispersion into account yielding more adequate standard errors of the estimates.[13]

The probability of survival after the end of pregnancy for all causes (within 42 days+late+sequelae related) of maternal death during 2013 was calculated by means of the Kaplan-Meier estimator: [14]

$$\hat{S}\left(t\right)=\prod_{t<T}\frac{n-d}{n}$$ (2)

Where *t* corresponds to the days elapsed between the end of pregnancy and death, *d* is the number of deaths occurred before time *T*, and *n* is the number of women whose pregnancy ended.

Finally, the degree of statistical dependence between the classification of a maternal death as late or sequelae-related, and causes mentioned in the death certificate (underlying and contributing causes) was assessed through a multiple cause mortality analysis of the BIRMM dataset. This approach provides information on statistical associations between underlying and contributing causes of death, revealing common combinations of events or conditions which lead to death.[15,16] For this analysis, we took into consideration all underlying and contributing causes listed on the death certificates.

The unit of analysis was each underlying and contributing cause entered on the death certificate; for example: if a death certificate for an individual woman lists one underlying cause and three contributing causes, such a woman would contribute with four units of analysis. Underlying causes of death were aggregated into six groups as ICD-MM recommended. The groups were pregnancy with abortive outcome; hypertensive disorders in pregnancy, childbirth, and the puerperium; obstetric hemorrhage; pregnancy related infection; other obstetric complications and indirect obstetric causes.[17] The relationship between each cause and a late or sequelae-related death was estimated by fitting logistic regression models to estimate odds ratios and 95% confidence intervals, with the form:

$$logit\left(p_{i}\right)=\beta_{0}+\beta_{1i}L\;\text{and}\;logit\left(p_{i}\right)=\beta_{0}+\beta_{1i}S$$ (3)

Where *L* it is an indicator variable that takes the value of 1 if the cause corresponded to a woman who suffered a late death and 0 if the cause corresponded to a maternal death that occurred within 42 days after end of pregnancy; p_i_ is the probability of a cause belonging to the i-th group. Similarly, S is an indicator variable which equals 1 if the cause corresponds to a death classified as a sequelae and 0 if the cause corresponds to a maternal death that occurred within 42 days after the end of pregnancy.

All analyses were performed in Stata version 13 (StataCorp, College Station, TX) and considered estimates with a p-value of less than 0.05 as being statistically significant.

### Ethical issues

No consent was obtained because only anonymized information obtained from clinical records and death certificates was analyzed. The study was approved by the Ethics Committee of the National Institute of Public Health of Mexico (approval number CI:1340).

## Results

**Fig 1:**
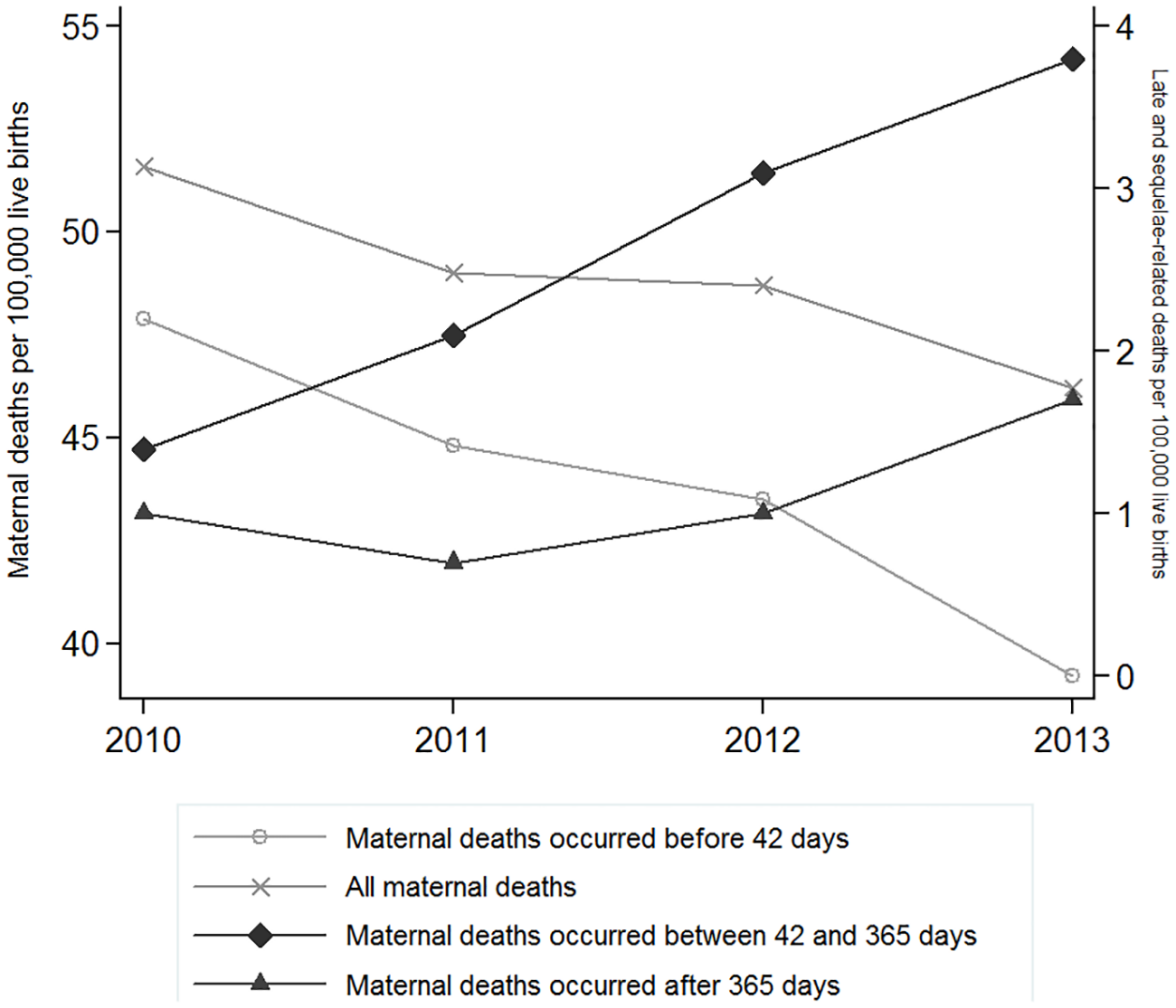
Trends of the Maternal Mortality Ratio by death categories (within 42 days, late and sequelae-related). Mexico 2010–2013.

During the study period (2010–2013), 4217 deaths related to pregnancy, childbirth and postpartum occurred in Mexico, of these 3,783 occurred within 42 days (89.7%), 227 were classified as late deaths (5.4%) and 207 were classified as sequelae- related (4.9%). Negative binomial regression showed that while the reported MMR declined at an average rate of 5% per year between 2010 and 2013, the MMR for late deaths increased by more than 100% going from 1.4 per 100,000 live births in 2010 to 3.8 per 100,000 in 2013 (p <0.01); sequelae-related deaths also showed an upward trend, although it was not statistically significant. However, when adding late and sequelae-related deaths to those that count for the government reported MMR, the rate of decline of the joint MMR is much lower, reaching only 3% per year (p = 0.015) (Fig 1). In total, the percentage of maternal deaths excluded from the MMR calculation doubled from 7.2% (77 deaths) in 2010 to 15.1% (153 deaths) in 2013.

**Fig 2:**
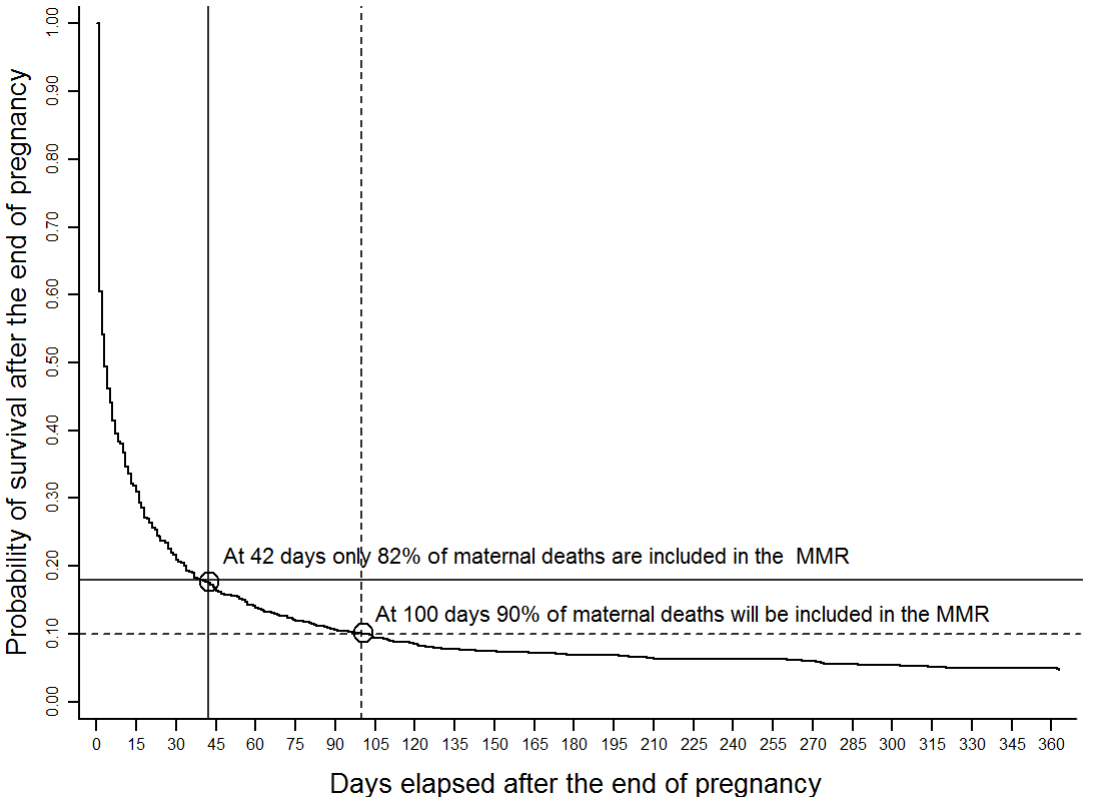
Probability of survival by days elapsed after the end of pregnancy; Kaplan-Meier survival function. Mexico, 2013.

The proportion of clinical summaries that included information on the date of the end of pregnancy increased with time; up to 30% of the summaries made in 2010 lacked this information, compared to 14% in 2013. The survival analysis was performed on the information of 1,013 deaths occurred in 2013; 128 (12.6%) of these deaths contributed with 0 time because they occurred before the end of pregnancy (they died while being pregnant) and 139 (13.7%) were not included because of a lack of identifiable information on the date of the end of pregnancy. The analysis revealed that no special behavior of the function (no abrupt drop or change) occurred around the cutoff at 42 days; it was found that most deaths occurred within the first 3 days after birth and then the survival function smoothly decays along the 364 days after birth. Over the whole 2010–2013 period, the 42 day-threshold caused approximately 10% of deaths to be excluded; however in 2013, 18% of all deaths related to pregnancy, childbirth and postpartum with available information on the time elapsed from the end of pregnancy to death, were left out of the calculation of the official MMR. Based on the Kaplan Meier survival analysis of 2013 data, we estimate that a hypothetical 10-week threshold would capture 87.5% of all deaths and a 100 day period would capture approximately 90% (Fig 2).

**Fig 3:**
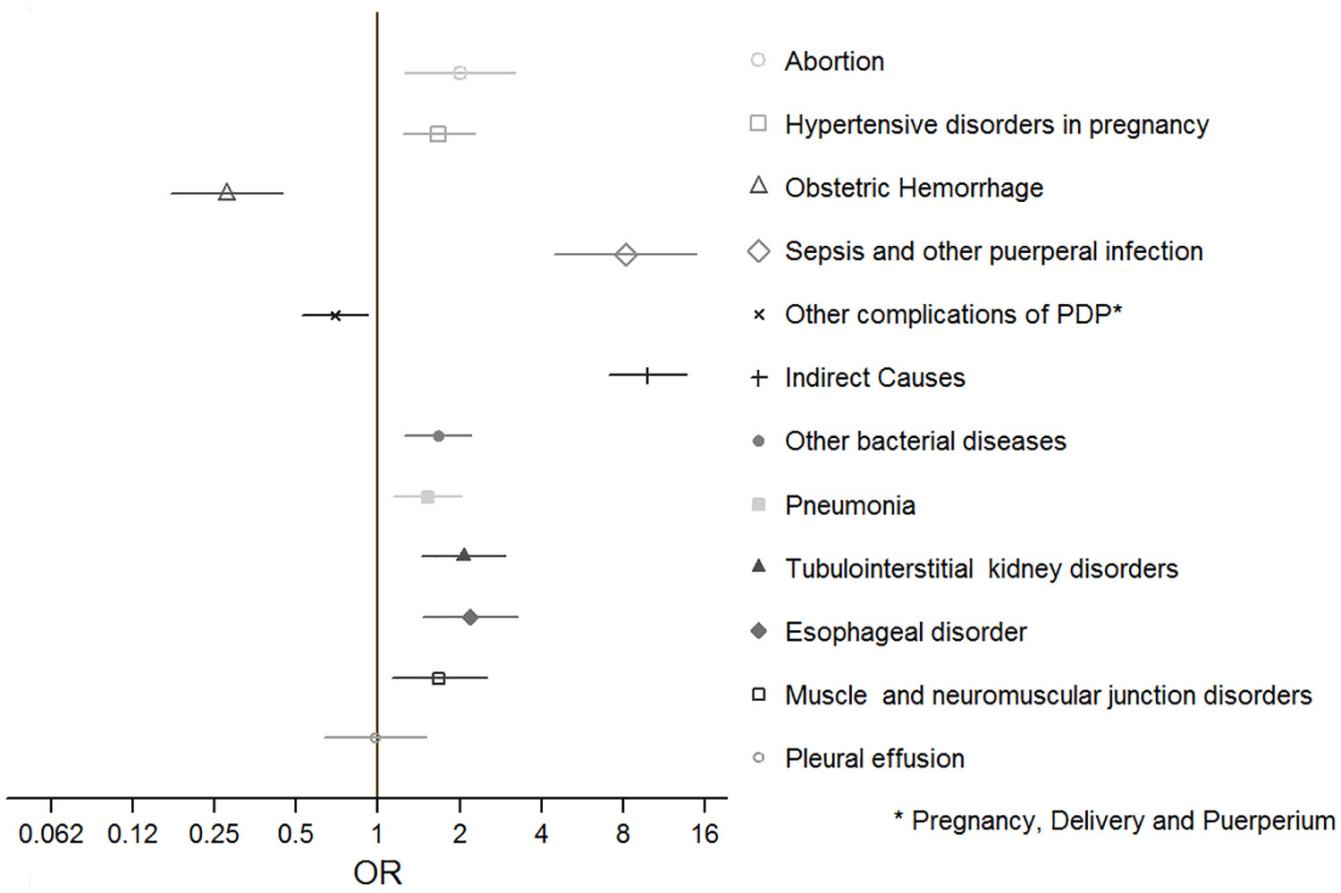
Odds ratios (OR) and 95% confidence intervals for causes related to late maternal deaths (deaths occurring after 42 days but before 365 days after the end of pregnancy). Mexico, 2010–2013.

**Fig 4:**
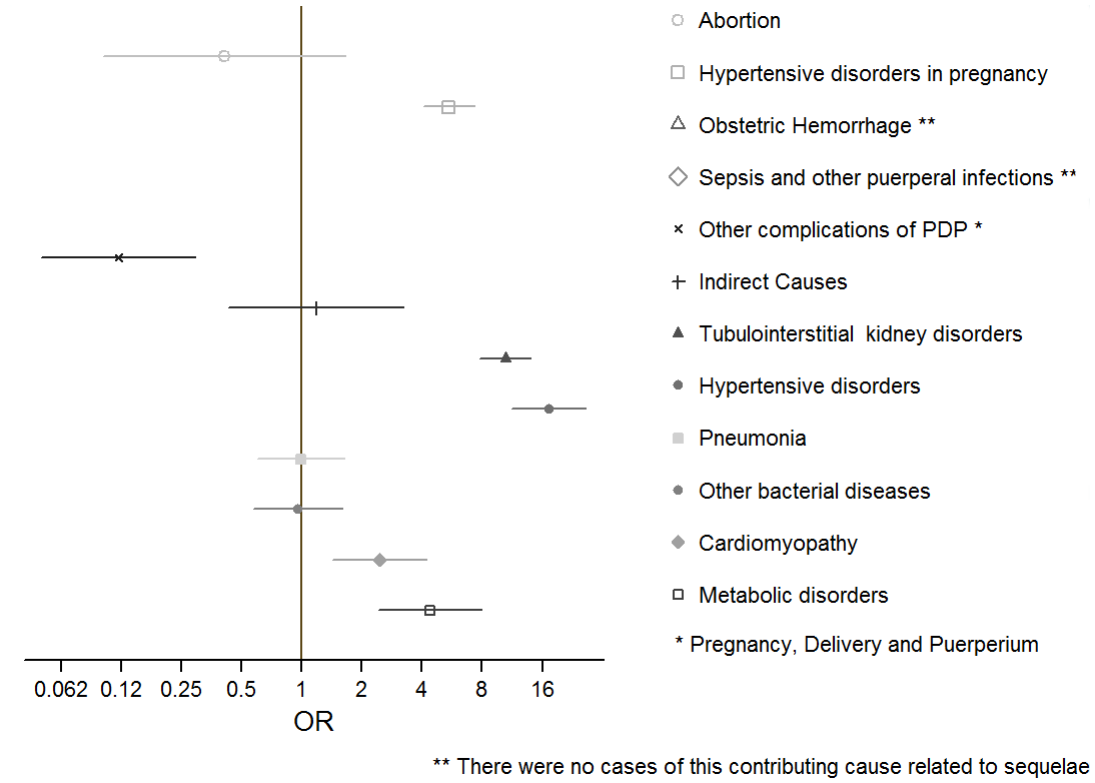
Odds ratios (OR) and 95% confidence intervals for causes related to deaths from sequelae of obstetric causes (deaths occurring 365 days or more after the end of pregnancy). Mexico, 2010–2013.

The multiple cause analysis showed a strong association between the excluded deaths (late and sequelae-related) and obstetric causes. Late maternal deaths had a greater chance of being associated with sepsis and other puerperal infections (ICD 10th O85-O86, OR = 8.1, 95% CI: 4.5–14.8) and with indirect obstetric causes (ICD 10th O98-O99, OR = 9.8, 95% CI: 7.1–13.6), than deaths occurred within 42 days. By contrast, obstetric hemorrhage (ICD 10th O20, O44-O46, O67, O72) was significantly inversely associated with the occurrence of a late death (OR = 0.28, 95% CI: 0.17–0.44) (Fig 3). Deaths classified as sequelae, were associated with hypertensive disorders in pregnancy (ICD 10th O10-O16, OR = 5.4, 95% CI 4.0–7.3), tubulointerstitial renal disorders (ICD 10th N16, OR = 10.4, 95% CI: 7.8–14.1) and hypertensive diseases not associated with pregnancy (ICD 10th I10, OR = 17.3, 95% CI: 11.3–26.6) (Fig 4).

## Discussion

Maternal death is fortunately becoming a rare event in Mexico, and all around the world it shows a very significant decline over the past twenty-five years. Estimates of the worldwide MMR decreased from 283.2 deaths per 100,000 live births in 1990 to 209.1 in 2013. [5] According to our results, in Mexico the official MMR decreased from 47.9 in 2010 to 39.2 in 2013. Yet paradoxically this decline is thwarted in some countries by a large increase in late and sequelae-related maternal deaths.[5] We would expect that, if we are being truly successful in combating maternal mortality, the trend of these deaths should accompany the decline of the official MMR as has indeed happened in India, China or the world average (late maternal death ratios for India, China and the World were respectively 3.5, 0.2 and 1.5 per 100,000 live births in 2000, compared to 2.0, 0.05 and 1.0 per 100,000 live births in 2013). [5] However, Mexico, as it is evident from our results, and other countries do not conform to this expected behavior, and it would appear as if many maternal deaths are not truly being avoided, but only postponed. We should be looking for plausible explanations of this paradoxical behavior of the data. Intuitively, we can think of reasons associated with improved registration and surveillance systems; and the increase in survival of patients with the appropriate use of medical technology; on the other hand there are reasons to suggest that by not being part of the inputs for the calculation of the official MMR there might be an undue "deliberate" accumulation of maternal deaths after 42 days of delivery. The pressure to do so could be released by relaxing the definition of maternal death time-wise. For instance, according with our analysis, extending the threshold to 100 days could include 90% of all maternal deaths in the MMR.

The United States of America is one of the countries that has increased its late and sequelae-related MMR, increasing from 2.2 per 100,000 live births in 2000 to 3.2 in 2013.[5] Lu et al state that the recent increase of maternal deaths in the US can be explained not only by improved registration but by a real change in maternal morbidity.[18] For these authors, the increase in maternal mortality is just the tip of the iceberg, because behind it there is a real change in epidemiological patterns of women who are currently pregnant (advanced age, higher prevalence of alcoholism, smoking, overweight, obesity, diabetes mellitus, etc.) which makes for a more severe and unwieldy maternal morbidity.

To advance our knowledge of late maternal deaths, the key variable to be analyzed is the date of delivery. Unfortunately, this variable does not appear on the death certificate and for that reason, we were forced to resort to clinical summaries in the BIRMM that describe some of the medical conditions that accompanied the maternal death. The elaborate reconstruction of this variable allowed us to estimate the time distribution of maternal deaths following childbirth, and from this, make proposals for a more inclusive definition of the MMR.

Knowledge about the timing of late maternal deaths and sequelae is enhanced by knowledge of the causes that accompany them; late maternal deaths appear to be mostly associated with infections (sepsis) or indirect causes. The fact that hemorrhage is inversely associated with late death shows that women with obstetric hemorrhage tend to die in the first hours after delivery, and have high chances of survival if they pass that critical period. Moreover, sequelae-related deaths are mainly associated with hypertensive disorders, either underlying and possibly aggravated by pregnancy, or due to pregnancy itself (preeclampsia/eclampsia), or to kidney disorders, which can in turn also be related to hypertensive disease.[19] Some of the women who died after 42 days may have actually been survivors of an obstetric near-miss; in a study from Burkina Faso, women who experienced a near-miss complication had a higher risk of all-cause and pregnancy-related death, even 4 years later, due to a combination of medical, social and health-care-related factors.[20]

Since the MMR is the indicator to monitor progress towards the Millennium Development Goal No 5A and will be used for one of the sub-goals of the Sustainable Development Goals #3, its definition and report is internationally uniform. However, we have reached the deadline for the MDGs, and this is perhaps a time to reflect on how to measure the maternal mortality phenomenon in a more accurate and fairer manner henceforth. Regardless of what is reported internationally, there are alternatives that can be used to improve the management of maternal health programs and achieve its ultimate goal of saving women’s lives. This has been practiced in countries who regularly conduct "Confidential Enquiries into Maternal Deaths" and report direct and indirect obstetric as well as incidental (deaths from unrelated causes which happen to occur during pregnancy or the puerperium), and late pregnancy-related causes.[21–25] The Centers for Disease Control and Prevention, has influenced state and national reports related to pregnancy to go beyond the causal definition of maternal death under ICD 10, defining a pregnancy-related death as “*the death of a woman while pregnant or within 1 year of pregnancy termination—regardless of the duration or site of the pregnancy—from any cause related to or aggravated by the pregnancy or its management*, *but not from accidental or incidental causes*”.[25] This is consistent with their pregnancy-related death surveillance system that began in 1987 and is capable of capturing 35% more maternal deaths than vital statistics.[26]

Rather than suggesting adapting the maternal mortality reports to the needs of each country, it is important to seriously reflect on the conceptual and ethical limitations of the current definition of the MMR. The temporary simplification of the MMR suggested by the MDG manual leads to a distortion of the true magnitude of the problem which operates in favor of a reduction of the indicator, but does not suit the purpose of accountability by not addressing the issue of maternal mortality in a comprehensive manner. According to our analysis of the Mexican situation in 2013, 18% of maternal deaths were excluded from the MMR due to the use of the 42-day threshold and, according to Kassebaum and colleagues in the US the exclusion is 17% and globally it is 15%.[5] Although the discussion to incorporate late maternal deaths to the MMR is not new, what is indeed relatively recent is the recommendation of the United Nations to foster a human rights approach to the implementation of policies and programs to reduce maternal morbidity and mortality.[27] This recommendation is based on the principles of equity and non-discrimination, and to be consistent with its spirit, the MMR should add all maternal deaths, focusing more on the fact that they happened, and less on nuances of *when* they happened. A more detailed discussion was set by Fukuda-Parr and Yamin, who added that the MMR is not the best indicator for measuring the progress of reproductive health from the perspective of human rights.[28,29] We believe that, if a decision to expand the definition of MMR to include late maternal deaths were to be made, no additional staffing or infrastructure would be needed. However, we would specifically recommend including a pregnancy checkbox on the standard death certificate as has already been done in the United States.[30]

In conclusion, our proposal is to change the construction of the MMR by including all late and sequelae-related deaths to its numerator; not only to count with a more comprehensive and useful indicator of the overall phenomenon of maternal mortality, but to present a complete account of maternal death and avoid unfair discrimination and exclusion of women that died as tragically as those who died within 42 days of giving birth.
